# Comment on Vidart et al. Relationship among Low T3 Levels, Type 3 Deiodinase, Oxidative Stress, and Mortality in Sepsis and Septic Shock: Defining Patient Outcomes. *Int. J. Mol. Sci.* 2023, *24*, 3935

**DOI:** 10.3390/ijms241813940

**Published:** 2023-09-11

**Authors:** I-Shiang Tzeng

**Affiliations:** Department of Statistic, National Taipei University, New Taipei 237303, Taiwan; istzeng@gmail.com

**Keywords:** sepsis and septic shock, monotone likelihood, methodology

## Abstract

In a previously published article, Vidart and colleagues conducted a prospective cohort study to explore the correlation between T3 levels and the risk of sepsis and septic shock in patients, with a follow-up period of 28 days or until deceased. The authors concluded that patients with sepsis and septic shock exhibited a significantly high risk of having low T3 levels. This research provides valuable insights into the clinical perspective in this particular field. However, certain clinical considerations should be addressed to enhance the study’s merit for researchers.

We are writing a comment on the article titled “Relationship among Low T3 Levels, Type 3 Deiodinase, Oxidative Stress, and Mortality in Sepsis and Septic Shock: Defining Patient Outcomes” by Vidart et al., published in the *International Journal of Molecular Sciences* in February 2023 [1]. The study aimed to investigate the levels of plasma type 3 deiodinase and its correlation with low T3 levels, as well as its impact on mortality and evolution to chronic critical illness (CCI) in patients with sepsis and septic shock. The authors identified low T3 levels as an independent prognostic factor in these patients, with low T3 levels at admission to the intensive care unit (ICU) being associated with a high risk of progression to CCI and mortality. The research is highly relevant with important clinical implications in the field. However, a more detailed analysis of the author’s interpretation of specific clinical considerations by the authors would enhance our understanding of the merits of the study.

In their analysis, the authors compared mortality between the T3 < 60 ng/dL and T3 ≥ 60 ng/dL groups, revealing a 4.89-fold increase in the adjusted hazard ratio (HR) for low T3 levels compared to the reference group among patients admitted to the ICU (survivors (*n* = 166); non-survivors (*n* = 94); adjusted HR = 4.89; 95% confidence interval (CI): 2.48–9.66). This raises concern regarding the influence of the specificity of the dataset on the estimated risk effect [2,3]. A broad representation of the CI of the HR as a *monotonic likelihood* hinders the confirmation of the true HR [2]. Thus, the authors could use the profile *penalized log likelihood* to modify the 95% CI, owing to the confidence in the HR [3]. Referring to the data in Table 2 of Vidart et al.’s study, it appears that inferring few cases associated with death within the T3 < 60 ng/dL group might have contributed to the sparsity of events, which raised the probability of *monotone likelihood*. To resolve this issue, data penalization along with an effective approach introduced in 2001 can be employed. Accordingly, a crude HR of (5 × 166)/(1 × 94) = 8.83 can be inferred and calculated based on our previous experience. To the best of our knowledge, the crude HR is frequently larger than the adjusted HR [4]. Subsequent to adjusting for T3, SAPS III, and Charlson, the eliminated effect was discovered with a narrower CI (adjusted HR = 4.89; 95% CI: 2.48–9.66). However, the current issue does not primarily focus on single-variable models [2]. When accessing multiple regressions, factors such as the number of participants and events, degree of association with the outcome, and size of the balance might affect the generality of the monotonic likelihood. Thus, the addition of a *monotone likelihood* limitation to the study of Vidart et al. is suggested, considering the sparse effect observed in the clinical data of patients admitted to the ICU.

## Data Availability

The data used to support the findings of this study are included within the article.
